# Phenotypic Antimicrobial Resistance Patterns in *E. coli*, *Salmonella*, and *Enterobacter* Species Isolated from Broiler Chicken Meat in Mzimba, Malawi: Insights from the Multiple Antibiotic Resistance Index

**DOI:** 10.3390/antibiotics15101028

**Published:** 2026-10-16

**Authors:** Abel Compbel Chipembo, Martin Henderson Kalumbi, Aminata Kamara, Naserah Fatmata Koroma, Marie Louise Iragena, Kumbukani Chikosa, Mary Maimu, Goliath Eneya Zulu, Precious Innocent Mastala, Sam Mvula, Thomas Malinki, Rejoice Msiska, Alberto Pondja, Janelisa Musaya, Belisário Moiane, Niura Madalena Bila

**Affiliations:** 1 Department of Animal Production and Food Safety, Faculty of Veterinary Sciences, Eduardo Mondlane University, Bairro Luis Cabral, Avenida de Moçambique, Km 1.5, Maputo P.O. Box 257, Mozambique; 2 Centre of Excellence in Agri-Food Systems and Nutrition (CE-AFSN), Eduardo Mondlane University, Praça 25 de Junho Posta 257 Edifício da Reitoria 5◦ Andar, Maputo P.O. Box 257, Mozambique; 3 Mzuzu Regional Veterinary Laboratory, Mzuzu P.O. Box 8, Malawi; 4 Department of Biomedical Sciences, Malamulo College of Health Science Campus, Malawi Adventist University, Makwasa P.O. Box 55, Malawi; 5 Department of Chemical Engineering, Faculty of Engineering, Eduardo Mondlane University, Avenida de Moçambique, Km 1.5, Maputo P.O. Box 257, Mozambique; 6 Department of Epidemiology and Biostatistics, Mel and Enid Zuckerman College of Public Health, The University of Arizona, 1295 N. Martin Ave., Tucson, AZ 85724, USA; 7 Malawi Liverpool Wellcome Trust, Chichiri, Blantyre P.O. Box 30096, Malawi

**Keywords:** antibiotic resistance, antimicrobial use, food safety, chicken meat, informal markets, Mzimba, Malawi

## Abstract

Background: Antibiotic resistance is becoming a serious problem in chicken production globally due to the use of antibiotics. In Mzimba, the multiple antibiotic resistance index (MARI) pattern in chicken meat is not known and has not been documented. Objective: To assess the prevalence of antibiotic-resistant *Escherichia coli*, *Salmonella*, and *Enterobacter* species in broiler chicken meat sold in informal markets in Mzimba District, Malawi, using the MAR index. Materials and Method: A cross-sectional study was conducted between March and May 2025. A total of 100 meat samples were collected from the informal markets across the district. A stratified sampling technique was used to select chicken meat samples from various markets. Descriptive statistics and inferential statistics were performed using R version 4.5.3. Results: *E. coli* (69%) and *Enterobacter* (12%) species were the most frequently isolated pathogens. The highest prevalence of antibiotic resistance was observed in *E. coli* against ampicillin (91%), meropenem (83%), and tetracycline (71%). Similarly, *Enterobacter* spp. showed substantial resistance to tetracycline (83%), ampicillin (83%) and meropenem (75%). The MAR index values were considerably high, ranging from 0.1 to 0.75. A total of 97.1%, 91.7% and 100% of the positive isolates of *E. coli*, *Enterobacter* and *Salmonella* spp., respectively, had MAR index values over 0.2. Location was the most significant predictor of the MAR index (*p* = 6.096 × 10^−5^, η^2^ = 0.372). Conclusions and Recommendations: High levels of the MAR index were revealed in this study. As such, combating MDR requires coordinated multisectoral approaches through One Health programs that integrate surveillance, policies, and innovation to prevent and control antimicrobial resistance.

## 1. Introduction

Antimicrobial resistance (AMR) is a pressing global health challenge with significant implications for environmental, animal, and human health [1,2]. According to the WHO, the ecosystem, animal, and human health are interlinked, and antibiotic-resistant pathogens may be shared among them [3]. Furthermore, imbalances in these relationships can lead to new infections that can be difficult to treat both in humans and in animals, leading to an increase in morbidity, mortality, and healthcare costs [4]. A significant proportion of human diseases originates from agricultural practices, such as poultry farming, which are associated with the development of antibiotic-resistant foodborne pathogens [5,6]. These pathogens are passed on to the ecosystem and to humans [7].

A study by [8] reported that antibiotics are used in food animal sources (FAS) worldwide due to the demand for animal protein and are frequently used in food animal production in developing countries in Africa to promote the well-being and growth of animals [9]. A study by [10] reported that in Africa, there is a high level of antimicrobial use, especially tetracyclines, aminoglycosides and penicillins in animal production systems. Such use can lead to antimicrobial resistance [11,12,13,14,15]. Several antibiotic-resistant foodborne pathogens have been isolated from human samples in developing countries, including Zambia, Mozambique, Tanzania, and Malawi, which are linked to increased morbidity and deaths [16,17,18]. For example, in Malawi in 2019, there were 3600 deaths attributable to AMR, and 15,700 deaths were associated with AMR worldwide [16]. The worst scenario is that it is estimated that AMR will be responsible for 10 million deaths per year globally by 2050 if appropriate interventions are not implemented to combat its spread [19,20]. This necessitates the estimation of the levels of antimicrobial resistance in foodborne pathogens in developing countries such as Malawi to inform appropriate policies and interventions. Unfortunately, most studies have targeted human samples; however, animals play a major role in antibiotic resistance [21]. To accurately combat AMR, there is a need to tackle the source of infections, especially farm animals, as evidence has shown that 60% of human infections originate from animals [3,5,21,22]. As such, estimating the burden of AMR in chickens, especially carcasses, could be one of the first steps in tackling AMR.

In Malawi, antibiotics are used as growth promoters, for disease prevention, and in the treatment of infections associated with unhygienic conditions and lack of biosecurity [23,24]. Although antibiotics are commonly used in Malawi in chicken production, efforts to enforce their regulation have had limited impact. There have been several attempts by the Malawi government to combat AMR by deploying extension workers and veterinarians in all districts to assist in prescribing antibiotics for animals and providing necessary information [25].

The government also developed treatment guidelines for poultry diseases in Malawi [26]. However, all these efforts have had limited impact due to inadequate funding, insufficient personnel, and a lack of socio-economic incentives [27]. Additionally, farmers purchase most of the antibiotics from informal market channels or unregistered veterinary shops whose owners are not qualified veterinarians or who do not know the pharmacokinetics and pharmacodynamics of antibiotics, while others use antibiotics obtained from hospitals for human infections [24,25,28,29]. Lack of awareness and knowledge regarding antimicrobial use and resistance, improper prescriptions, and the use of antibiotics intended for human medicine can lead to incorrect concentrations and sometimes sub-therapeutic dosing, facilitating the development of AMR in bacteria that cause infections in chickens [10,23,24,30,31]. For instance, antibiotic residues have been reported in meat residues [32]. Exposure to low doses of antibiotics facilitates the development of AMR in bacteria that cause infections in chickens [33,34]. Recent studies in Malawi have reported the presence of antibiotic-resistant pathogens in broilers; for example, the authors of [34] reported resistance profiles of *E. coli* to ampicillin, cefotaxime, ciprofloxacin, gentamicin and sulfamethoxazole trimethoprim based on broiler fecal samples, and the authors of [35], using chicken rectal swabs and chicken droppings, also recorded high resistance rates of *E. coli* to tetracycline, gentamycin, erythromycin and ampicillin. Moreover, a national survey on AMR in broiler chicken reported 115 *E. coli* strains from 116 fecal and cecal samples from broiler chickens across Malawi that were resistant to tetracycline (90%) and co-trimoxazole [36]. These resistant pathogens, which carry resistance genes, are passed on to humans directly via contact with animals, feces, food of animal origin, and the environment [3,37]. Most of the antibiotics to which pathogens develop resistance are commonly used in both animals and human in Malawi [24,28,38].

The improper use of antibiotics in chicken farming is driven by the growing demand for poultry products in Malawi as a source of animal-based proteins [39,40]. As such, farmers have resulted to non-prudent antimicrobial use on farms, with the intent to optimize growth and prevent and treat poultry diseases for profit maximization [23,24,31]. Mzimba District is one of the districts in Malawi where this practice presents a growing challenge due to the high demand for chicken meat, as it is less costly than other meat types. In this area, chicken meat is sold as a whole or cut into pieces, and consumers buy the parts they prefer. Chicken meat is mostly sold in informal or formal markets where the enforcement of food safety standards and microbial testing facilities are lacking. The informal markets in this area are also characterized by unhygienic conditions that can lead to further contamination of the meat by different foodborne pathogens such as *E. coli*, *Salmonella*, and *Enterobacter* species.

It was therefore the aim of this study to assess the prevalence of antibiotic resistance of *E. coli*, *Salmonella*, and *Enterobacter* species in chicken meat sold in informal markets using the multiple antibiotic resistance index (MAR index) tool. This was the first study to assess the prevalence of antibiotic resistance of the three selected bacteria in chicken meat from informal markets in Mzimba using the MAR index tool. This could help in providing evidence-based interventions such as the development of food safety policies, enforcing guidelines for prudent antimicrobial use in food animals, protecting vulnerable populations from hard-to-treat infections, and understanding One Health dynamics to prevent the spread of antibiotic resistance.

## 2. Results

### 2.1. Prevalence of E. coli, Salmonella *spp.*, and Enterobacter *spp.*

**Figure 1:**
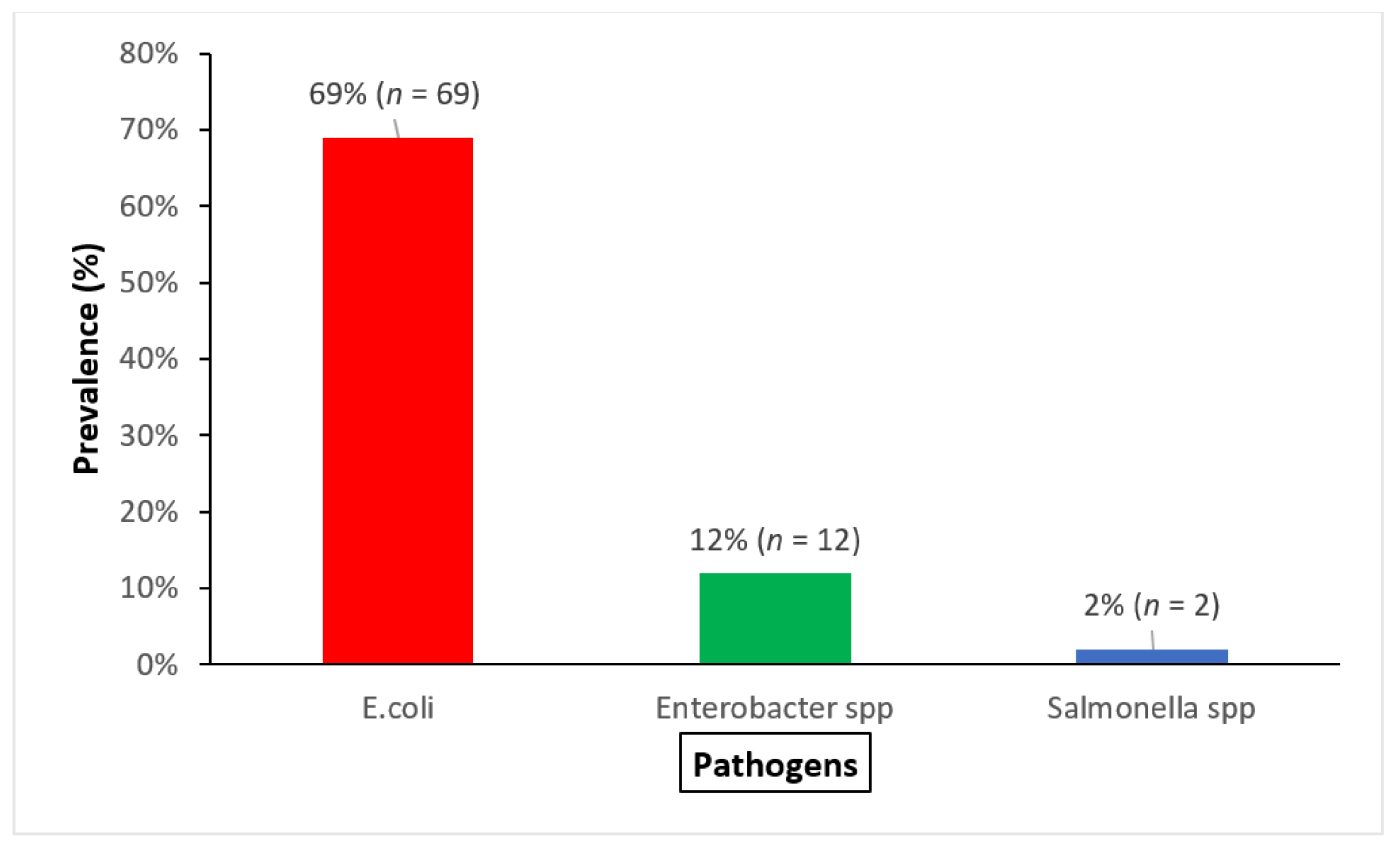
Prevalence of *E. coli*, *Enterobacter* pp., and *Salmonella* spp. in chicken meat.

The results of the overall prevalence of *E. coli*, *Salmonella*, and *Enterobacter* species are presented in Figure 1. Out of the collected 100 samples, the prevalence of *E. coli* was high, followed by *Enterobacter* spp. and then *Salmonella* spp.

**Figure 2:**
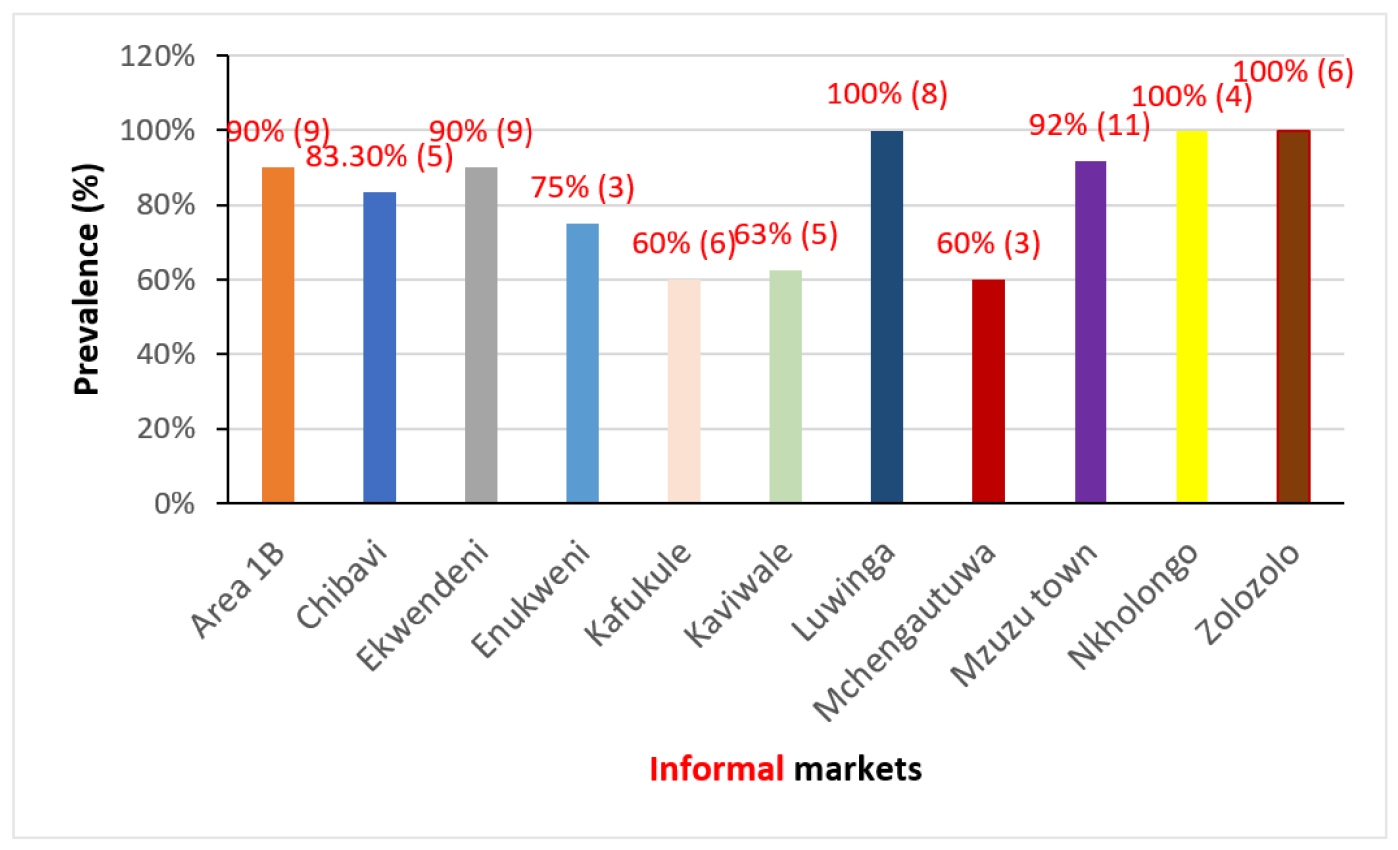
Prevalence of *E. coli* by informal markets.

**Figure 3:**
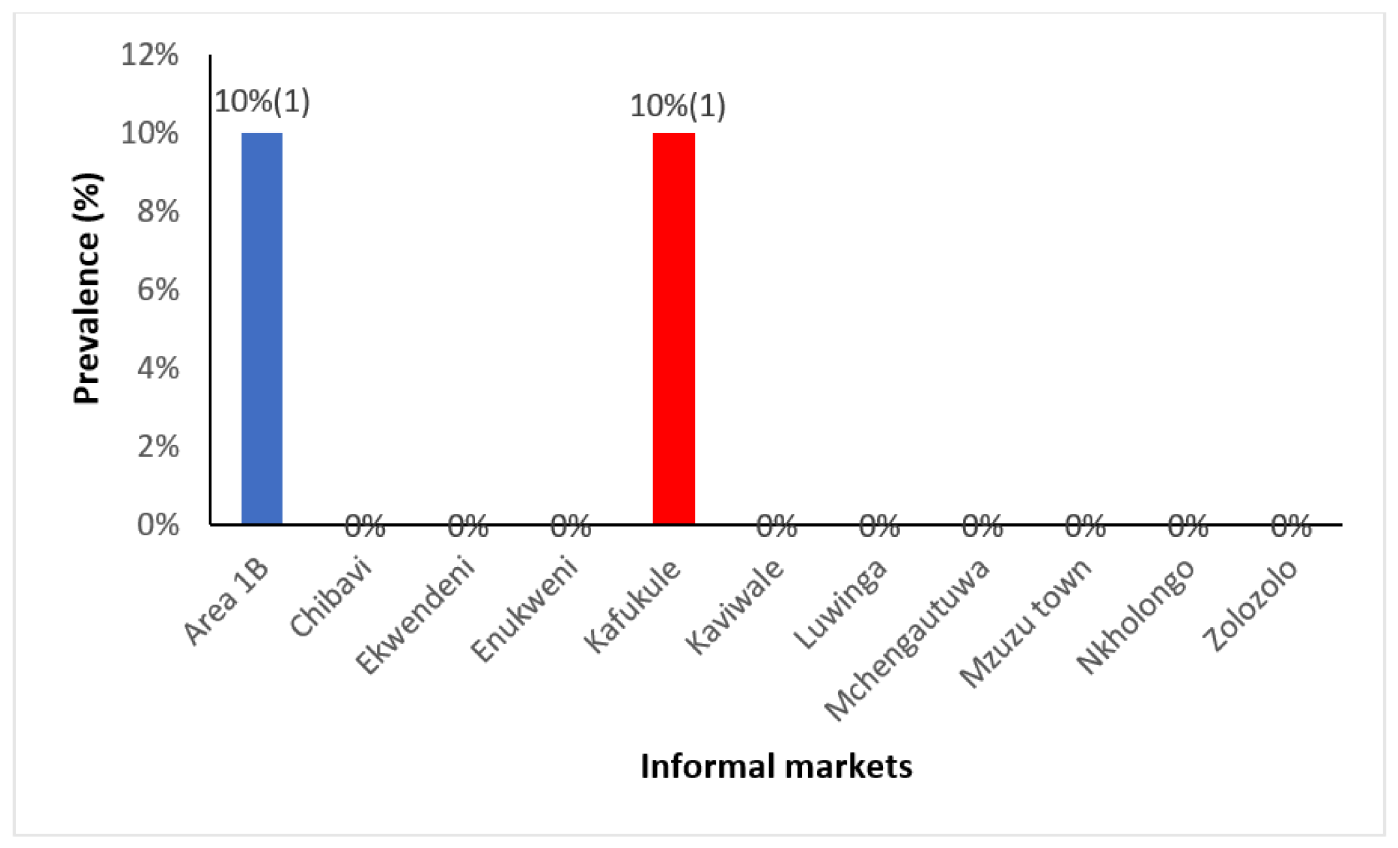
Prevalence of *Salmonella* species by informal markets.

**Figure 4:**
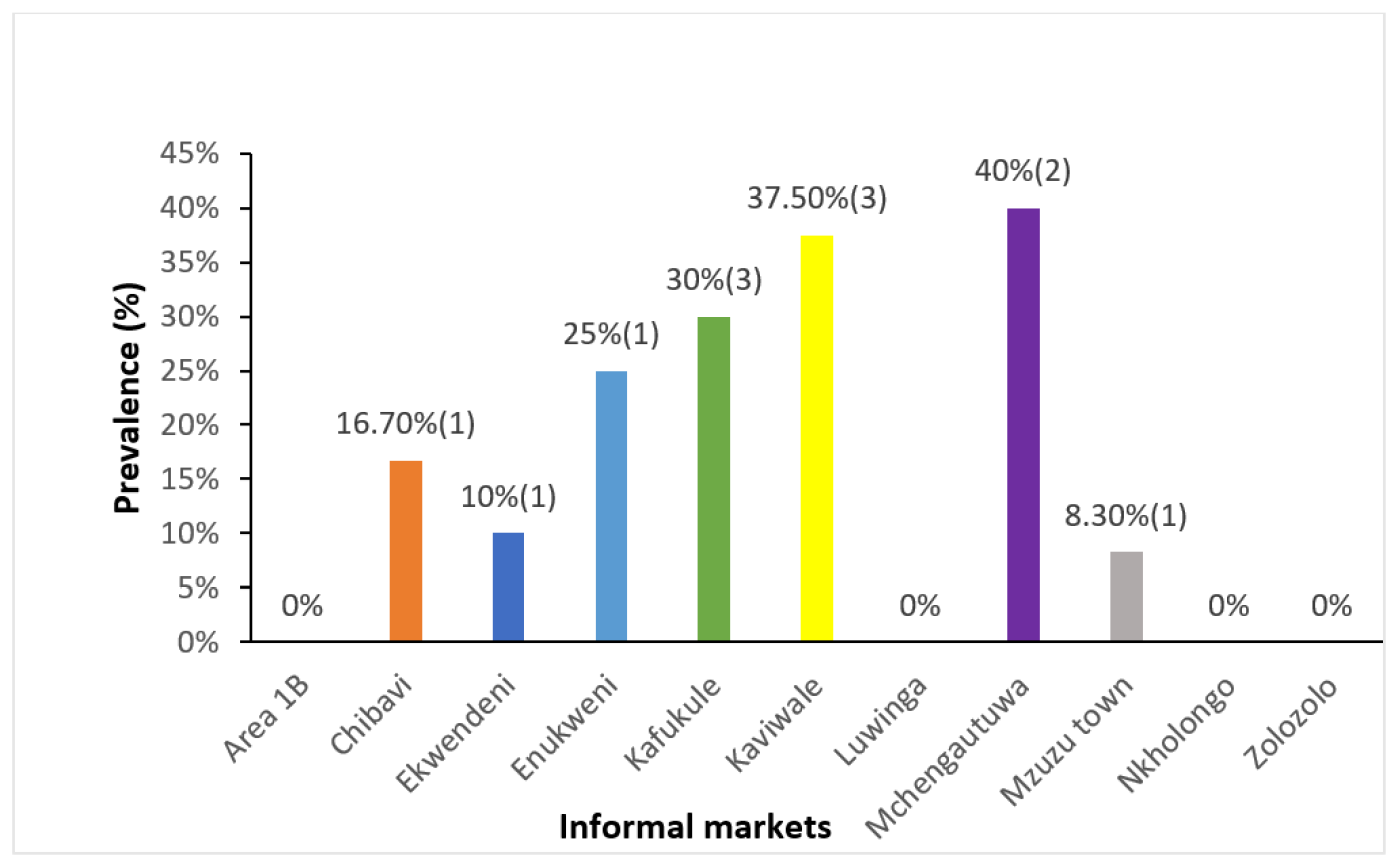
Prevalence of *Enterobacter* species by informal markets.

When the results were stratified by sample collection location, *E. coli* was observed to be the most prevalent pathogen across locations, ranging from 60% in Mchengautuwa and Kafukule to 100% in Luwinga (*n* = 8), Nkholongo (*n* = 4), and Zolozolo (*n* = 6), followed by *Enterobacter* species with the highest prevalence of 40% (n = 2) in Mchengautuwa (Figure 2). The least prevalent pathogens by location were *Salmonella* species, which were isolated from two sites only (Figure 3), namely, Area 1B and Kafukule (Figure 3). Of all the locations, Kafukule was the only one in which all the pathogens were isolated (Figure 4).

### 2.2. Prevalence of Antibiotic Resistance in E. coli, Salmonella, and Enterobacter Species

**Figure 5:**
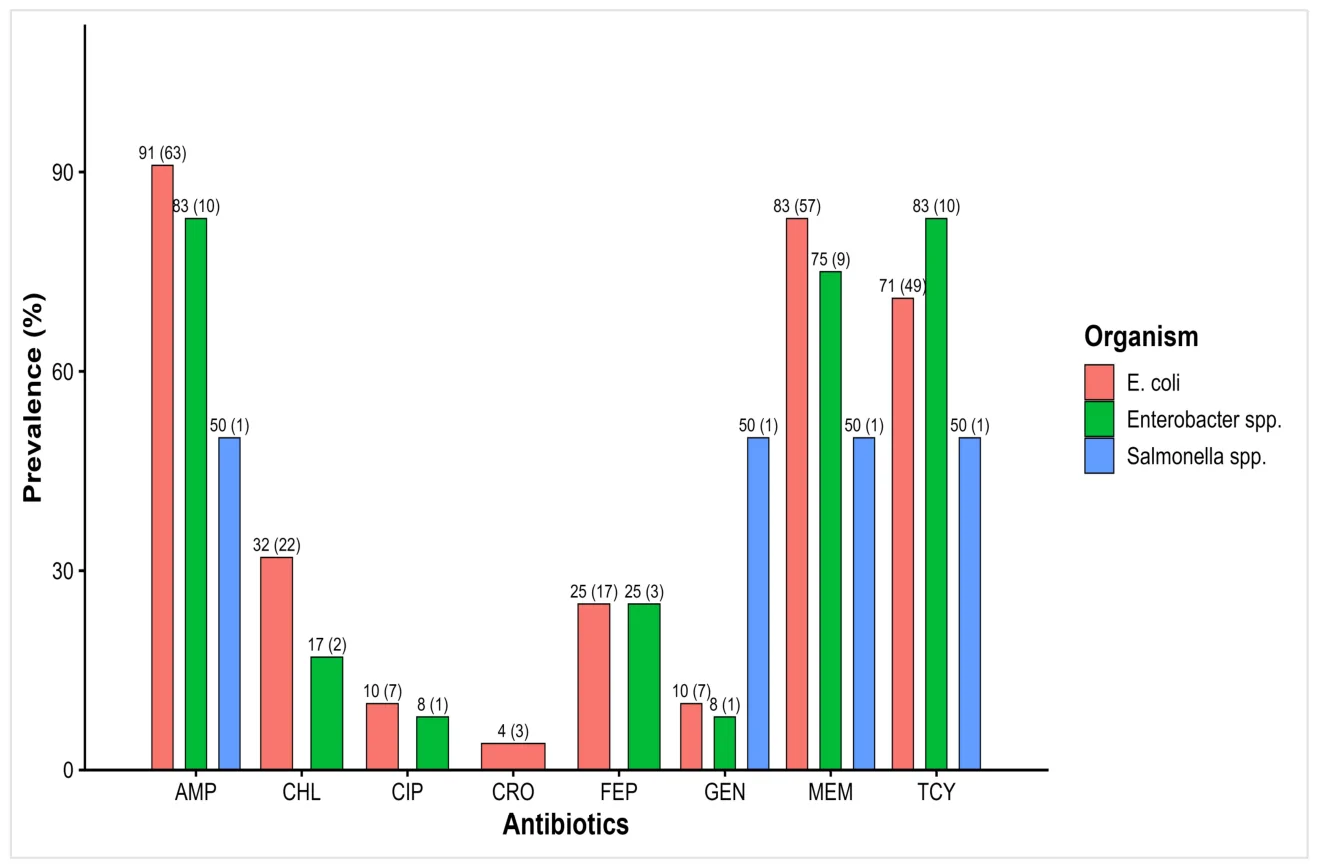
Prevalence of antibiotic resistance in *E. coli*, *Salmonella*, and *Enterobacter* species against selected antibiotics. AMP = ampicillin, CHL = chloramphenicol, CRO = ceftriaxone, FEP = cefepime, GEN = gentamicin, MEM = meropenem, TCY = tetracycline. Key: Percentage (counts/n).

The *E. coli*, *Salmonella*, and *Enterobacter* species isolated from broiler chicken meat samples showed substantial resistance against the tested antibiotics (Figure 2). The highest occurrence of resistance was observed for *E. coli* against ampicillin (91.3%, n = 63), followed by *Enterobacter* species against ampicillin (83.3%, n = 10) and tetracycline (83.3%, n = 10). Other substantial resistance was seen for *E. coli* against meropenem (82.6%, n = 57) and tetracycline (71%, n = 49). For *Salmonella* species, the highest levels of resistance of 50% (n = 1) were recorded against ampicillin, gentamicin, and meropenem antibiotics (Figure 5).

#### 2.2.1. Prevalence of Antibiotic Resistance by Location

The results of the prevalence of antibiotic resistance by location are presented in Table 1. It was observed that the majority of the isolates from nearly all the study sites were resistant to ampicillin (AM), meropenem (MEM), and tetracycline (TCY). Across all study sites, the resistance to ampicillin ranged from 73% to 100%, meropenem from 33% to 100% (except at Kafukule), and tetracycline from 33% to 100%. However, some isolates across the study sites demonstrated substantial susceptibility to chloramphenicol, gentamicin (GEN), ciprofloxacin (CIP), ceftriaxone (CRO), and cefepime (FEP).

#### 2.2.2. Multiple Antibiotic Resistance Index of *E. coli*, *Salmonella*, and *Enterobacter* Species

**Figure 6:**
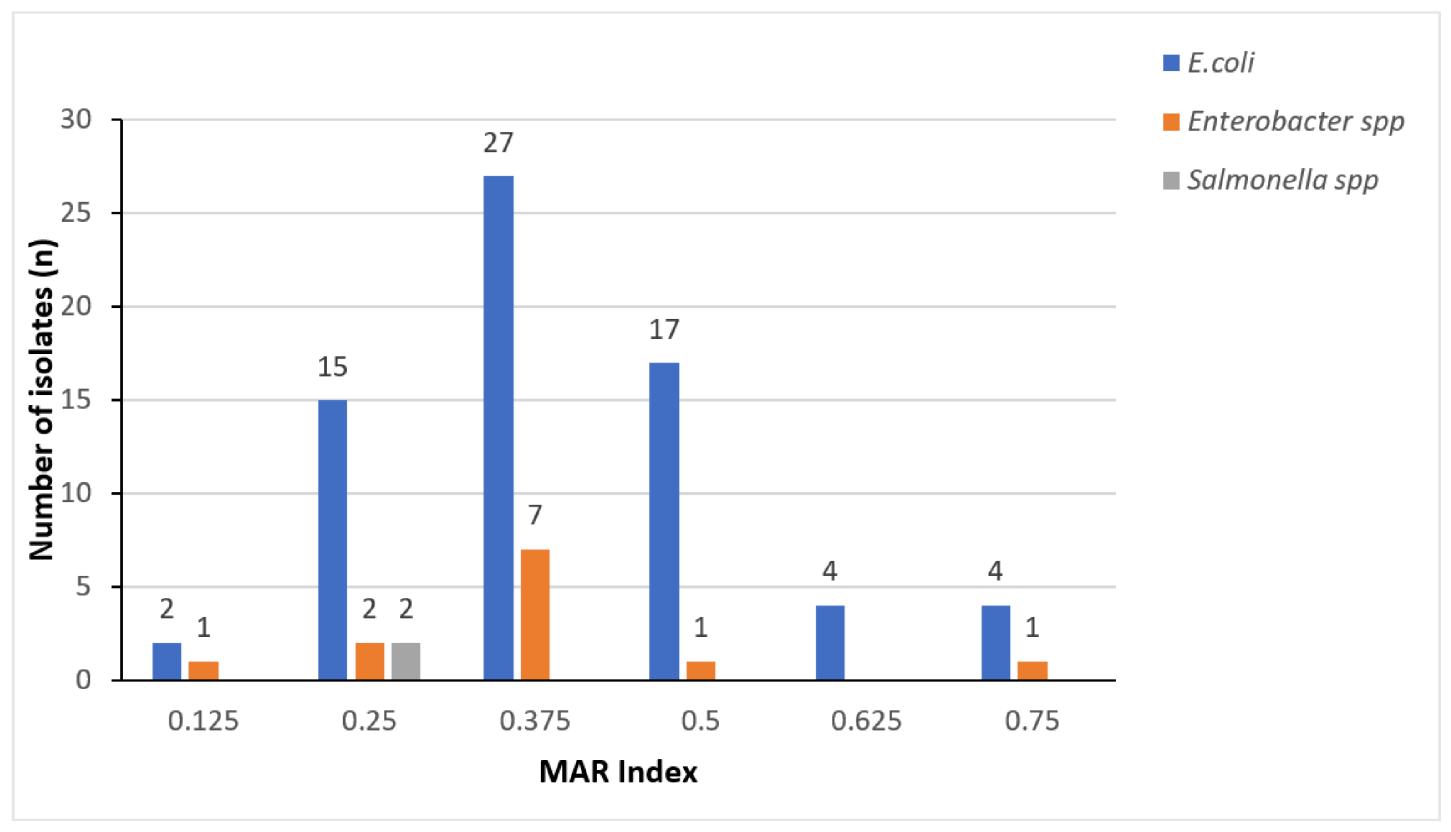
The occurrence of multiple antibiotic resistance across organisms.

The multiple antibiotic resistance index (MAR index) was determined by dividing the number of antibiotic-resistant isolates by the total number of antibiotics tested, and the results are presented in Figure 6 and Supplementary Material Tables S1 and S2 [41]. A very high multiple antibiotic resistance index of 0.75 was observed in 5.8% (n = 4) of *E. coli* and 8.3% (n = 1) of *Enterobacter* species. This study revealed that the highest occurrence of multiple antibiotic resistance was 39.1% (n = 27) observed with the MAR index of 0.375, followed by 24.6% for the MAR index of 0.50 and 21.7% for the MAR index of 0.25 in *E. coli* pathogens. While the most common occurrence of multiple antibiotic resistance (8%) for *Enterobacter* species was observed with the MAR index of 0.38. Overall, 97.1%, 91.7% and 100% of the positive isolates of *E. coli*, *Enterobacter* spp and *Salmonella* spp., respectively, had MAR index values of over 0.2. When the MAR indices were compared across study sites, they were found to be significantly different (*p* = 0.001).

#### 2.2.3. Effect of Organism Type and Location on MAR Index

To determine whether organism type influences the MAR index, the Kruskal–Wallis test was applied and revealed that organism type had no significant effect on the MAR index (*p* = 0.1376) (Table 2). The location where the samples were collected was the dominant predictor that significantly influenced the MAR index (*p* ˂ 0.0001), with a large effect size (η^2^ = 0.372).

To identify individual significant locations and organisms, beta regression was performed; a sensitivity analysis showed that the median remained stable under all sensitivity testing methods, indicating the robustness of the results (Table S5). The regression results revealed that Enukweni, Kafukule, Kaviwale, Luwinga, Nkholongo, Zolozolo, and Mchengautuwa had significantly lower MAR index values compared to the reference location Area 1B. Although overall organism type did not significantly influence the MAR index in the Kruskal–Wallis test, beta regression demonstrated that *Salmonella* species had a significantly lower MAR index compared to the reference *Enterobacter* species (Table 3).

Type of estimator: ML (maximum likelihood). Log-likelihood: 70.34 on 14 Df. Pseudo R-squared: 0.4351. Number of iterations: 23 (BFGS) + 2 (Fisher scoring). Number of iterations: 23 (BFGS) + 2 (Fisher scoring).

**Figure 7:**
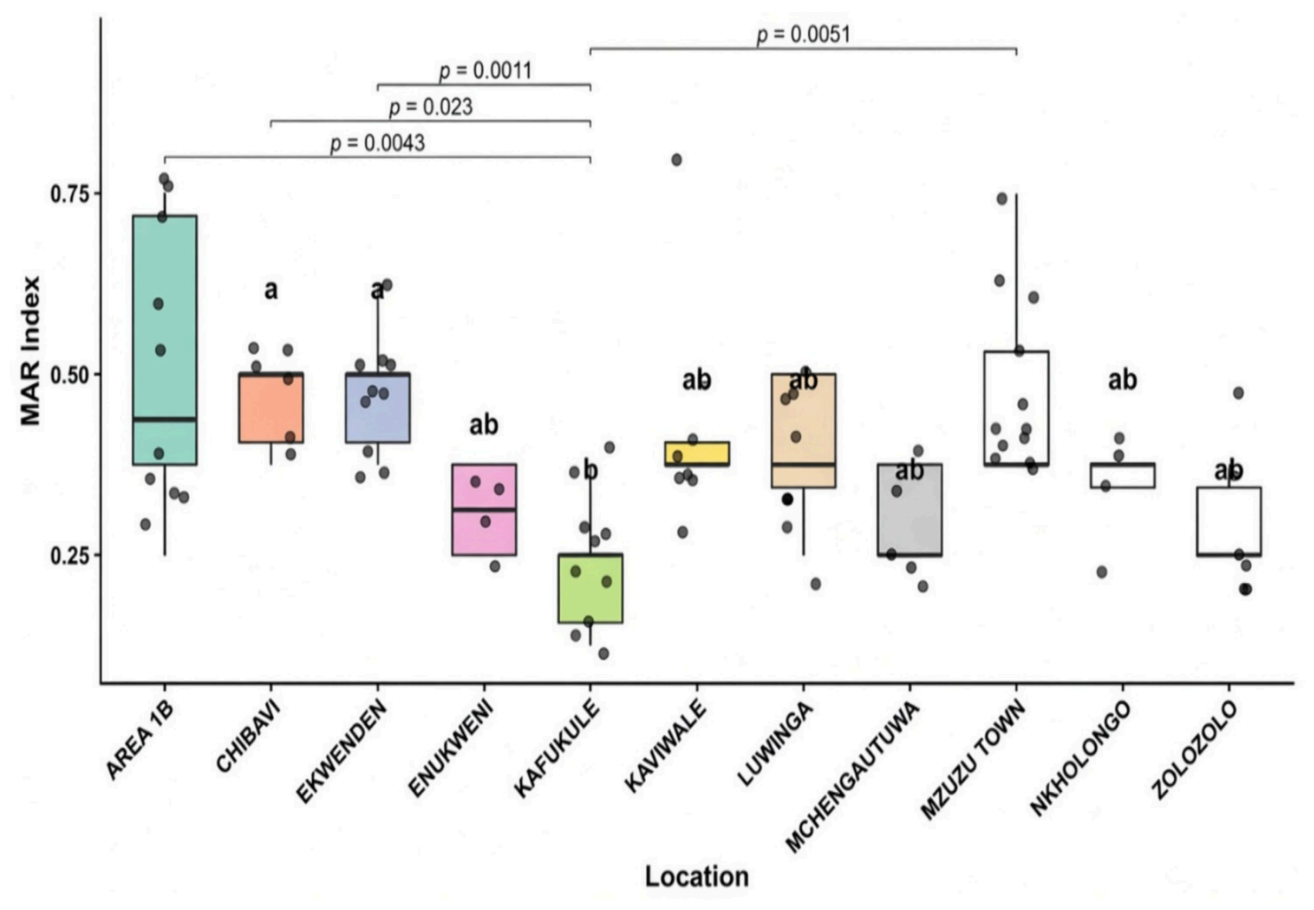
Pairwise comparisons of multiple antibiotic resistance index by location. Locations with similar Dunn letters (e.g., a or ab) do not have statistically different MAR indices.

Post hoc Dunn’s test with Bonferroni correction was performed for pairwise comparison of locations since location was significant, and the results revealed that Kafukule’s MAR index differed significantly from those of Area 1B (0.0043), Chibavi (*p* = 0.023), Mzuzu Town (*p* = 0.0051), and Ekwendeni (*p* = 0.0011). It was also observed that locations such as Chibavi and Ekwendeni had similar MAR indices (*p* > 0.05). Similarly, Enukwenu, Kaviwale, Luwinga, Mchengautuwa, Nkhorongo, and Zolozolo had similar MAR indices (*p* > 0.05). (Figure 7; Table S3).

## 3. Discussion

### 3.1. Prevalence of E. coli, Salmonella, and Enterobacter Species

The prevalence of *E. coli*, *Salmonella*, and *Enterobacter* species observed in this study could be attributed to the improper use of antibiotics in broiler farming, which leads to the genetic modification of antibiotic targets on these pathogens to ensure survival and selection. These findings could also be associated with fecal contamination of meat due to poor hygiene, as reported by others [42]. Additionally, these organisms may be present in chicken meat as a result of contact with contaminated hands or the environment, especially fecal-contaminated water used for cleaning the meat. The ruptured digestive tract causes *E. coli*, which was initially present in the digestive tract, to contaminate chicken meat [43]. It was evident during this study that vendors were not maintaining adequate levels of hand washing when slaughtering and processing chickens, thus increasing the levels of bacterial contamination, as the contents of the digestive tract could come into contact with the meat. These findings agree with Tresse in France, who reported that chicken meat is easily contaminated by meat handlers during handling and processing [44]. When the results were compared with the literature, it was evident that the prevalence of microorganisms can be affected by numerous factors beyond hygienic practices. For instance, the lack of biosecurity in chicken production, environmental exposure, and contaminated feed given to chickens can affect the prevalence of microorganisms [45,46,47,48,49,50]. However, the high prevalence of *E. coli* observed in this study implies that hygienic practices were not optimal in these areas, necessitating further enforcement. These findings are very concerning compared to the levels reported in Indonesia (57%) and South Africa (66.8%) [51,52]. These variations depict the high levels of unhygienic practices in the informal markets in Malawi that require further improvements and strict regulations, such as standardized slaughtering, hygiene, and storage practices. However, similar findings were reported in Germany, implying that meat contamination by pathogens is a wider problem affecting different countries [53].

On the contrary, it was surprising to note that the occurrence of *Enterobacter* species in broiler chicken meat was low (12%) compared to *E. coli*. These organisms are predominantly associated with gut colonization and unhygienic environments [54,55]. These low levels of *Enterobacter* species suggest that, despite unhygienic practices, the organism may not be ecologically dominant in the study area, or that the survival and growth characteristics and processing factors do not favor it. These levels of *Enterobacter* species concur with the findings reported by Amer and Schwaiger in Egypt and Germany [42,56], who reported prevalences of 20.8% and 22%, respectively, which are higher than the findings of 6.9% in Turkey [57]. Although the occurrence is low, these pathogens have public health significance, especially in immunocompromised individuals, particularly in Sub-Saharan Africa, where the rates of diabetes and HIV infections are high. In Malawi, the prevalence of diabetes is 5.7%, and HIV is 7% [58,59], indicating that the majority of people can be adversely affected by these organisms through the food chain. Other than *Enterobacter* species, the occurrence of *Salmonella* species was very low (0.02%), suggesting that it is also not ecologically predominant in this area. Similarly, low levels (0.005%) were reported in Zambia, indicating a regional occurrence of *Salmonella* in broiler chicken meat [60]. The prevalence of *Enterobacter* spp. and *Salmonella* spp. has limited statistical comparison with the studies indicated due to the low number of samples that were positive for these two pathogens. The reliance on biochemical tests alone, with no molecular confirmation (e.g., 16S rRNA sequencing) for organism-level identification, especially for *Enterobacter* spp. differentiation from other Enterobacteriaceae might also contribute to the low prevalence observed in the study.

### 3.2. Prevalence of Antibiotic Resistance

This study revealed a high occurrence of antibiotic-resistant *E. coli*, *Enterobacter*, and *Salmonella* species in the meat, which is a serious public health concern. These bacteria include extended-spectrum beta-lactamase (ESBL) producers, which are capable of hydrolyzing antibiotics such as penicillins and cephalosporins [61]. High resistance can be due to the overuse or improper use of antibiotics, causing bacteria to develop resistance mechanisms to protect themselves against the effects of the drugs by developing cell membranes that block the entry of antibiotics into bacterial cells [62]. Genetic modification is often associated with incorrect drug selection, injection duration, and dose, which eventually lead to treatment failures [63]. Resistance can also be due to the transfer of resistant genes from the environment near chicken cages, as most vendors were using unclean cages for different chickens. It was observed that buyers could touch the cage and that the cages were also placed on the dirty ground, which could cause the transfer of organisms across environments. Previous studies conducted in Brantyre and Lilongwe showed that farmers use antibiotics for growth promotion and to treat chicken infections by using antibiotics received from public hospitals [12,24,38]. Usually, these antibiotics are given to chickens in low dosages or inappropriate dosages, leading to the development of resistance. This is perpetuated by the lack of knowledge, attitudes, and practices on antimicrobial use and resistance by farmers in Mzimba [24]. Misuse of antibiotics in chicken farming may lead to a natural imbalance in the microflora in chickens, leading to a decrease in bacterial sensitivity to antibiotics [64]. The high levels of antibiotic resistance observed in this study are concerning, as antibiotic-resistant pathogens are commonly transmitted to humans through the food chain.

Apart from antibiotic use, cross-contamination might also contribute to the antibiotic resistance in meat because high microbial loads on unclean surfaces such as knives, cutting boards, and scaling drums can transfer resistant strains of bacteria such as *E. coli*, *Enterobacter* and *Salmonella* species from one chicken to another. This is because visible cracks, rust, or meat residues on knives or cutting boards can promote biofilm formation and microbial proliferation. Pathogens such as resistant *E. coli* and *Salmonella* species attach themselves to these surfaces by forming an extracellular matrix that prevents them from being cleaned and increases the possibility of causing cross-contamination, as reported by others [65,66]. Inadequate hand hygiene and lack of gloves among vendors can also cause human- and environment-resistant pathogens such as *E. coli*, *Salmonella*, and *Enterobacter* species on meat, thereby causing the resistance patterns observed in this study.

The current findings show that a significant amount of resistant bacteria are present in chicken meat. Among the antibiotics tested, the pathogens were highly resistant to ampicillin (91.3%), suggesting the use of this antibiotic in chicken farming in Malawi. This agrees with studies performed in India and Bangladesh, but the rates in India and Bangladesh were much higher, 100 and 94%, respectively [67,68]. On the contrary, Seo and Lee reported high levels of ampicillin resistance of 75% in *E. coli* in Korea, signifying that antibiotic resistance in chickens is a global problem [69]. *E. coli* and *Enterobacter* species isolates also demonstrated substantial ampicillin resistance. This could be just the tip of the iceberg; there could be several pathogens resistant to ampicillin or antibiotics in the penicillin group. Further research is needed to comprehensively assess the susceptibility of different foodborne pathogens to ampicillin in this area.

The prevalence rates of *E. coli* resistance to meropenem were 82.6%, and *Enterobacter* spp. resistance to meropenem was 75%; thus, it was lower than the *E. coli* resistance. This could be attributed to the treatment of chickens with meropenem or using it as a growth promoter. This can also be due to cross-contamination with meat, horizontal gene transfer from *E. coli* infection in humans, and/or mobile chicken cages used to keep chickens during sale, as well as its common usage in chicken farming [24]. This resistance could also be attributed to the fact that most farmers keep their chickens inside the main house, where people sleep. This is a common practice in Malawi, where people keep chickens in houses to protect chickens from thieves; such poor biosecurity may contribute to meropenem resistance in *E. coli* and *Enterobacter* species through the exchange of pathogens between people and chickens, consistent with observations in a companion survey [24]. Additionally, co-selection could be another reason attributed to the development of resistance in *E. coli* and *Enterobacter* spp. to meropenem due to the chemicals farmers use as disinfectants, which contain metals, and such resistant metals can coexist with *E. coli* and *Enterobacter* spp., leading to meropenem resistance [70,71,72,73].

The high levels of resistance observed to gentamicin, chloramphenicol, ceftriaxone, and cefepime can be associated with the improper use of these antibiotics in this area or can be due to the lack of proper ways to discard these expired antibiotics in this area, which can contribute to *E. coli*, *Enterobacter*, and *Salmonella* spp. resistance [10,31,74]. In this area, farmers either use expired drugs, or, for those who discard expired drugs, they bury them in the ground; others throw them in a waste bin (dug in the ground), polluting the environment and consequently contributing to the development of resistance genes [73]. This suggests that One Health is a serious problem in this region. The authorities, specifically regulators, must ensure that the cross-transfer of these pathogens is broken through a strict One Health approach that integrates surveillance, policy, and innovation [75].

Although studies acknowledge the lack of oversight in informal markets and the role that these markets play in the rural economy, it is particularly important to incorporate sustainable approaches that are acceptable to local people to ensure that AMR is defeated. Although the current study has successfully identified meropenem resistance in poultry, future studies should consider triangulating the assessment of AMR and antibiotic use in humans, animals, and the ecosystem in this area. Otherwise, meropenem resistance is a serious public health issue because this antibiotic belongs to the carbapenem group, which is used in the treatment of human skin and stomach infections in adults and children as a last resort to fight bacterial infections [76,77]. According to the WHO, meropenem is classified as one of the high-priority antibiotics, and studies in developed and developing countries have reported the emergence of *E. coli* and *Salmonella* species resistant to meropenem in the food chain, consistent with our findings [78]. However, resistance in this study was identified phenotypically; future studies should focus on the detection of carbapenemase production or other resistance pathways.

It is concerning that resistant pathogens are difficult to treat, and they pose serious risks of disease transmission, serious illness, disability, and death. The improper use of selected antibiotics also has the potential to make antibiotics in the same class, or that have similar mechanisms of action, ineffective, thereby limiting the selection of antibiotics in human treatment [46]. For instance, Egocin is widely used in Malawi for treatment and as a growth promoter in poultry; it contains oxytetracycline, which promotes resistance to similar drugs such as tetracycline. High levels of tetracycline in *Enterobacter* species might be attributed to the use of Egocin and tetracycline.

Ciprofloxacin and gentamicin resistance were low and could be due to the increased use of quinolones and neomycin, respectively, in poultry farms. Neomycin is also one of the commonly used growth promoters and antibiotics used in Malawi [12]. It has a structure similar to gentamicin and contributes to gentamicin resistance. Similar levels of antibiotic resistance were reported by others in Malawi, Korea, and India, with levels ranging from 69 to 90%, highlighting a global problem and the need for multinational collaborations [36,79,80]. This global issue becomes more complex when these pathogens gain resistance to multiple antibiotics. Others across the world have reported that some strains of bacteria isolated from chickens displayed multidrug resistance, which is associated with the improper use of antibiotics in the poultry industry [81,82,83,84].

### 3.3. Multiple Antibiotic Index (MAR Index)

To understand antibiotic resistance better, one of the recommended surveillance methods, the multiple antibiotic resistance (MAR) index, was employed. In this study, most of the isolates for specific pathogens had an MAR index above 0.2, which is very concerning. An index above 0.2 suggests that almost all the samples were collected from high-risk areas. This MAR index threshold indicates that pathogens in chickens in these areas are exposed to selective pressure, which promotes multiple antibiotic resistance [85]. Other pathogens, particularly *E. coli*, revealed extreme MAR indices of 0.63 and 0.75. Therefore, the selection of antibiotics for the treatment of animal and human pathogens in this area may not be easy, as indicated by the MAR index values. To combat *E. coli* or *Enterobacter* infections in this area, evidence-based treatment should be a priority in both animals and humans. Antibiotic susceptibility testing should be a priority before prescribing antibiotics due to the recorded high levels of the MAR index and multidrug resistance (MDR). It should be routinely and comprehensively performed to assess the levels of resistance in other pathogens targeting all the antibiotics used in human treatment, as people in this area use antibiotics aimed at treating human infections in poultry [24]. Furthermore, coordinated or multi-sectoral surveillance programs such as integrated One Health surveillance need to be instituted; if present, they should be strengthened in public health and agriculture to halt the rising incidence of multidrug resistance.

This study demonstrated that sampling location is the most important predictor of the MAR index, unlike the bacterial species, with a Pseudo R-squared of 0.43. For environmental or biological data, this strongly suggests that location and organism explain a substantial portion of the variance in resistance. Area 1B exhibited a significantly higher MAR index compared to Kafukule, Enukweni, Ekwenden, and Mzuzu Town, while the Kafukule study site showed the lowest MAR index. This implies that the profiles of antibiotic resistance are driven by external factors such as the environment and farm management practices rather than heterogeneity across species. In this study, some locations showed significantly higher MAR indices (*p* < 0.05) compared to others, signifying the power of variation due to practices associated with farming and markets, such as antimicrobial use, marketing channels, and hygienic practices, among others. Studies have documented that unregulated antibiotic use is associated with increased MAR indices [86]. This notion supports this study that some locations are reservoirs of multidrug-resistant bacteria.

*Salmonella* species had a significantly lower MAR index compared to the reference *Enterobacter* species; this could be because the antibiotics used in this study were effective against *Salmonella* spp. and that, in the study location, they are not widely used to treat *Salmonella* infections in animals or humans and thus do not contaminate the meat, unlike in *Enterobacter*-associated infections. However, these claims should be interpreted with caution due to the small subgroup size of *Salmonella* incorporated in the beta-regression analysis. Therefore, this study recommends that the significance levels reported herein for organism-specific association should be treated as exploratory, requiring further studies with a larger number of *Salmonella* isolates to improve uncertainty.

Previous studies conducted in this area identified unregulated and improper use of antibiotics in broiler chickens in these areas [24]. These findings suggest that co-exposure of bacteria to a similar environment leads to similar patterns of antibiotic resistance. As such, to combat antibiotic resistance, interventions must not be bacteria-specific but rather should focus on environmental controls, mostly in high-risk locations. These findings align with the One Health framework that recognizes the role of human, animal, and environmental interactions in the spread of antibiotic-resistant bacteria [87]. For instance, the reported high levels of multiple antibiotic resistance in the current study are associated with poultry; the resistant bacteria can be shared with humans through contact or the food chain, and the waste from animals and humans carrying resistant strains can be shared with the environment. Similar patterns of the MAR index have been reported in multiple types of bacterial isolates from the environment, poultry farms, chicken droppings, and meat for various antibiotics in different countries, indicating a serious global One Health problem [88,89,90,91,92,93].

Although this study was well executed, it has limitations, especially those that are associated with the generalization of the findings. For instance, this study was conducted in one district, and it was limited to broiler chickens. As such, generalizations arising from this study should be taken with caution. However, the results of this study may offer a baseline comparison with other districts in Malawi and with other chicken breeds. This study did not assess meat quality and the One Health approach of integrating meat, environmental, and human samples (vendors’ hands) in assessing the relationship in terms of levels of bacteria and AMR. Similarly, this study did not attempt to assess the presence of antibiotic residues in broiler chicken meat and resistance genes, which could help in explaining the reasons for the high MAR index values. The absence of molecular confirmation of isolated pathogens and the lack of appropriate quality control strains for *Salmonella* and *Enterobacter* spp might also significantly affect the reliability of the results. Additionally, the use of existing laboratory protocols for sample analysis are also limitations of this study. The small prevalence of *Enterobacter* spp. and *Salmonella* spp. might also affect the statistical power. The lack of a stomacher to process the meat samples might also affect pathogen identification. Therefore, future studies should aim at assessing the prevalence of antibiotic residues and antibiotic resistance genes such as carbapenemase-producing genes (e.g., blaNDM, blaKPC) in bacteria isolated from chicken meat, as the current study lacks genotypic data. Future studies should also consider correlating the bacterial counts to check the quality and antibiotic resistance. Furthermore, comparing the antibiotic resistance profile among the environment, animals, and human beings should be a future focus.

## 4. Materials and Methods

### 4.1. Study Area and Period

**Figure 8:**
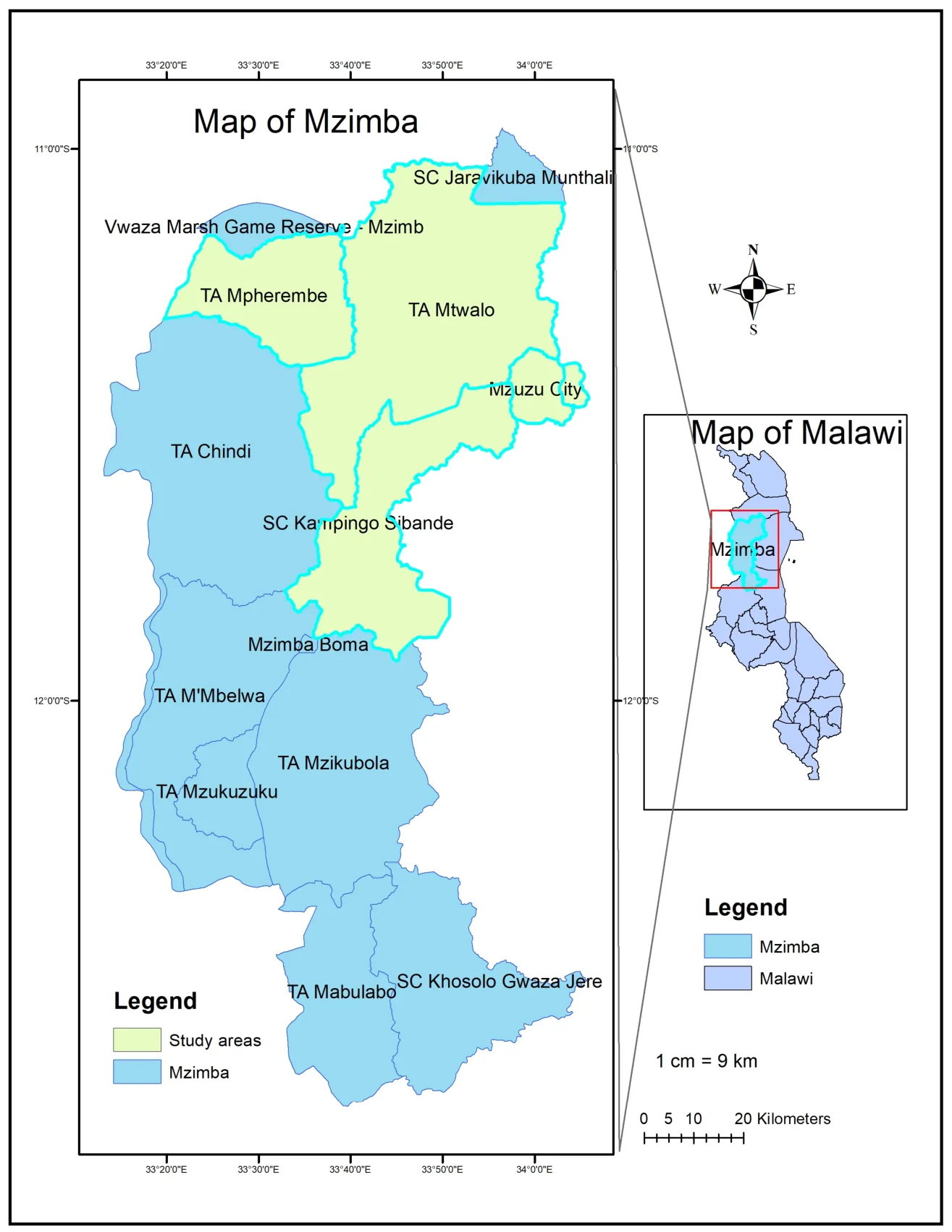
Map of Malawi showing the location of Mzimba District in green (on the **right**) and study areas in yellow. The map was drawn using ArcGIS Version 15 Software (Esris, Redlands, CA, USA).

This study was conducted in the northern region of Malawi, Mzimba District (Figure 8), targeting informal markets from 4 March 2025 to 21 May 2025. Mzimba District is located at a latitude of 11° 54′ 0″ S and longitude of 33° 36′ 0″ E. This study was conducted from March to May during the rainy season; the rainy season promotes bacterial multiplication and survival in meat samples. This district covers an area of 10,430 km^2^ and has a population of approximately 610,944. This study was conducted in the following informal markets: Enukweni, Ekwendeni, Kafukule, Mzuzu town, Chibavi, Mchengautuwa, Zolozolo, Area 1B, Nkholongo, Luwinga, and Kavibale. These areas have a notably large population of small-scale chicken farmers and vendors who sell chicken meat, and the area has been reported to lack awareness in terms of antibiotic use [24]. Most people come to these areas to buy chicken meat as a source of protein because it is less costly. At the same time, these areas act as trade centers where people can buy goods in bulk, making them a suitable catchment area for sample collection.

### 4.2. Study Design, Study Participants, Sampling Method, and Sample Size

A cross-sectional survey was conducted in the informal markets of Mzimba District. The sample size was calculated using OpenEpi, version 3 (www.openEPI.com), a tool designed for epidemiologic statistics. It was calculated using the formula below:

*n* = [DEFF × Np (1 − *p*)]/[(d^2^/Z^2^_1−α/2_ × (N − 1) + *p* × (1 − *p*)]

where

n: sample size
DEFF: Design effect: 1.0;
N: population size: (the total number of vendors selling/slaughtering chicken in Mzimba North);
d: confidence limits as % of 100 (absolute +/−%: 5%);
*p* hypothesized % frequency outcome in a population: 50%+/−5;
α (alpha): 0.05;
Z = 1.96.

OpenEpi was used to ensure that each chicken meat sample collected from each vendor had an equal chance of being selected and to ensure generalization of the results to the population. Using OpenEpi, the total sample size calculated for the study was 97, while the study population (total number of chicken vendors) was 124 according to the sampling frame of vendors known to sell chicken; however, 100 samples were collected. Potential participants were identified with the help of the market chair in each market. Purposively, stratified and random sampling were used in this study. The markets were purposively selected based on the number of vendors who sell chicken meat. The stratified sampling technique was used to select the vendors from which the meat samples were obtained. In this technique, each market was considered an individual stratum comprising the accessible population of vendors, and individuals were selected from each stratum using simple random sampling. In simple random sampling, a lottery method was used to select the vendors by giving them number codes, and then a person was masked (blind folded) to pick a paper by chance until the required number was reached. Finally, the meat samples were randomly collected from those vendors. From each vendor, only one chicken sample was collected. Since the vendors could sell chicken specific from one farmer, the sample collected could represent all chickens that are supposed to be slaughtered during a particular time and could represent the flock/farmer from where the chicken was obtained.

### 4.3. Inclusion and Exclusion Criteria

#### 4.3.1. Inclusion Criteria

The sampling frame comprised all the fresh broiler chicken meat samples collected immediately after slaughter in the informal markets. All fresh broiler chicken meat collected during slaughtering was included.

#### 4.3.2. Exclusion Criteria

Chicken meat sold in shops in bulk or retail was not included in the sample. Local breeds of meat chickens and layers were also excluded from this study.

### 4.4. Sample Collection

Sample collection was conducted in accordance with ISO standard 17604:2015 [94]. Aseptic techniques were used through the use of gloves to collect approximately two grams of chicken meat using forceps and a sterile surgical knife while ensuring that the blade was replaced between samples to prevent cross-contamination. Additionally, a sterile swab was used to collect samples by swabbing all internal parts and neck regions of the carcass following slaughter [94]. Both meat and sterile swabbed samples were kept in 30 mL of peptone water in a container as a pooled sample. The use of 30 mL of peptone water was based on the laboratory protocol. Then, the samples were transported to the Mzuzu Veterinary Laboratory in an isothermal box with ice packs, maintaining a temperature of less than 4 degrees. The temperature was monitored by a thermometer throughout the sampling, transporting, and laboratory sample processing stages. In the laboratory, the samples were left in peptone water enrichment media for 24 h in an incubator and were processed after 24 h [95].

### 4.5. Sample Processing, Bacterial Isolation, and Identification

#### 4.5.1. Quality Control

Quality control for the incubator, fridge, and biosafety cabinet was checked in terms of temperature before, during, and after the experiments. All media used were prepared according to manufacturer’s instructions. Positive and negative quality controls (QCs) of all the culture media used in this study were performed during media preparation and incubation to check media performance and sterility (contamination). This included documenting the culture medium name, brand, color, gross appearance, texture, and quantity of the weighed powdered media. Then, the appropriate amount of water was measured, and the pH of the broth was assessed. After media preparation, QCs were performed to verify the physical appearance, including the absence of wrinkles and precipitates, and to confirm sterility. For quality assurance of *E. coli*, the strain *E. coli* ATCC 25922 was used as a QC reference organism, while for *Salmonella* and *Enterobacter* species, previously isolated organisms were used as reference organisms but were not fully assigned a code. All internal QC procedures were conducted in accordance with the CLSI 2022 guidelines [96].

#### 4.5.2. Sample Preparation

Each meat sample, along with a carcass swab, was placed in 30 mL of peptone water (BPW) in a Falcon tube and incubated overnight at 37 °C. The use of buffered peptone was adopted from [60,97].

#### 4.5.3. Isolation of *E. coli*

All *E. coli* isolates were identified with guidance from practical food microbiology and ISO 7251:2005 [98,99]. Aseptic techniques were followed while isolating *E. coli*. Each enriched sample was inoculated on MacConkey agar (Becton, Dickinson and Company, New York City, NY, USA) adopted from Mulinga et al. [60] using a sterile loop and incubated at 37 °C for 24 h. Positive colonies, identified by their pink and yellow coloration, were subcultured onto MacConkey agar and incubated under the same temperature for an additional 24 h. For identification, colonies with pink–red coloration on MacConkey agar underwent microscopic characterization via Gram’s staining [100]. This was followed by seven biochemical tests, which included the Triple Sugar Iron (TSI), catalase, indole, oxidase, lysine, growth in urea, and Simon citrate tests. Colonies that exhibited gram-negative rods with red color, acid slant/acid butt in the TSI test with gas production (+) and no hydrogen sulphate production, and were indole positive as described in [98], catalase positive, urease negative, oxidase negative, lysine decarboxylation positive and Simmons citrate negative were regarded as *E. coli*.

#### 4.5.4. Isolation of *Salmonella* spp.

All isolates for Salmonella were identified with guidance and some modifications from practical food microbiology and ISO 6579-1:201 [97,99]. Aseptic techniques were followed during the isolation of *Salmonella* spp. Each enriched sample was inoculated on Xylose lysine tergitol 4 (XLT4) (MOLTOX^®^, North Carolina) adopted from [101] using a sterile loop and was incubated at 37 °C for 24 h. Positive colonies, identified by blue–green to blue with black centers coloration, were subcultured onto XLT4 and incubated under the same temperature for an additional 24 h. For identification, colonies with pink–red coloration on MacConkey agar underwent microscopic characterization via Gram’s staining. After the microscopic examination, a series of biochemical tests were performed, including the Triple Sugar Iron (TSI) test, motility, indole, oxidase, lysine, growth in urea, Simmons citrate, and catalase tests. Colonies that exhibited gram-negative rods, alkaline slant/acid butt in the TSI test with hydrogen sulphate production and were indole negative, catalase positive, urease negative, oxidase negative, lysine decarboxylation positive and Simon citrate positive were regarded as *Salmonella* spp.

#### 4.5.5. Isolation of *Enterobacter* Species

All samples for *Enterobacter* spp. were identified with guidance from practical food microbiology [99]. Aseptic techniques were followed during the isolation process. Each enriched sample was inoculated on MacConkey agar and Xylose lysine tergitol 4 (XLT4) agar adopted from [60,101] using a sterile loop and was incubated at 37 °C for 24 h. Positive colonies, identified by pink coloration and yellow on MacConkey agar and (XLT4) agar, respectively, were subcultured onto MacConkey agar and Xylose lysine tergitol 4 (XLT4) agar. For identification, colonies that were round and pink on MacConkey agar and round and yellow on XLT4 underwent microscopic characterization via Gram’s staining [100]. This was followed by biochemical tests, which included the Triple Sugar Iron (TSI), urea, indole, oxidase, lysine, growth in urea, Simmons citrate, and catalase tests. Colonies that exhibited gram-negative rods with red color, acid slant/acid butt in the TSI test with gas production (+) and no hydrogen sulphate production, and that were indole positive, catalase positive, urease negative, oxidase negative, lysine decarboxylation positive and Simmons citrate positive were regarded as *Enterobacter* spp.

#### 4.5.6. Antibiotic Susceptibility Testing (AST)

The Kirby–Bauer disk diffusion method is recommended for antimicrobial testing [96]. This method was used for the antimicrobial susceptibility testing of *E. coli*, *Salmonella* spp., and *Enterobacter* spp. The AST was conducted on Mueller–Hinton agar according to the guidelines of Clinical and Laboratory Standards 20222 [96]. All isolates were evaluated for antimicrobial susceptibility using the following antibiotics: ampicillin (10 µg), ceftriaxone (30 µg), chloramphenicol (10 µg), cefepime (30 µg), meropenem (10 µg), gentamicin (10 µg), and tetracycline (5 µg and 10 µg). The selection of these antibiotics was based on their priority classification, considering their importance for humans and animals [102], as well as their significance in animal production [103]. Additionally, the availability of these antibiotic types in the Mzuzu testing laboratory was considered.

The procedure for AST was adopted from Phiri [33]. A single colony was picked from a pure culture of *E. coli*, *Salmonella* spp., and *Enterobacter* spp. agar plates and suspended in 0.9% saline water, adjusting the solution to match a 0.5 McFarland turbidity standard. A volume of 0.1 mL from the 0.5 McFarland suspension was then swabbed evenly in at least three directions on Mueller–Hinton agar plates. After allowing each plate to dry, antimicrobial disks were placed at a specific location on the agar plates for further testing. The disks were placed 24 mm apart by using a disk dispenser and gently pressed down onto the agar surface to ensure uniform contact. The inoculated plates were then incubated at 37 °C for 18 to 20 h. After incubation, each plate was examined, and the diameter of the zone of inhibition was measured to the nearest whole millimeter with a measuring clipper ruler against a non-reflecting background. For *E. coli* and *Enterobacter* spp., the results were interpreted and categorized as Sensitive (S), Intermediate (I), or Resistant (R) according to the CLSI interpretation table [96] (Table 4).

For *Salmonella* spp., the results were interpreted and categorized as Sensitive (S), Intermediate (I), or Resistant (R) according to the CLSI interpretation table [96] (Table 5).

#### 4.5.7. Data Management and Analysis

Data from the laboratory study were entered into an Excel file, cleaned, and analyzed using R statistical software, version 4.5.3 (R Core Team). Descriptive statistics were used to summarize the data on the prevalence and resistance patterns among the tested microorganisms (*E. coli*, *Salmonella*, and *Enterobacter* species) in the form of frequencies and percentages. Inferential statistics were performed to compute pairwise comparisons and the effect of location and organism on the MAR index. Before analyzing the MAR index, the data were assessed for normality assumption using the Shapiro–Wilk test and visual inspection of the Q-Q plot (Table S4; Figure S1). Then, the Kruskal–Wallis test was performed to determine the overall effect of location and organism type. Pairwise comparisons of the MAR index across study sites were performed using post hoc Dunn’s test with Bonferroni correction to adjust for multiple testing. Eta-squared ((η^2^) was used to calculate effect sizes, which were interpreted as small (<0.06), moderate (0.06–0.14), or large (>0.14).

A beta regression model was fitted to assess the relationship between the MAR index (dependent variable) and location and organism (independent variables/predictors). The analyzed parameters were estimated using the logit link function. Beta regression was selected because the assessed dependent variable was a continuous variable bounded between 0 and 1. However, there was no MAR index equal to 0 or 1; as such, boundary adjustment transformation was not considered in this study. Additionally, the beta regression model was preferred over generalized linear models (GLMs) because it is suitable for modeling proportional data with heteroscedasticity and asymmetry, such as the MAR index data [104,105].

The multiple antibiotic resistance (MAR) index was determined for each isolate by using the formula MAR = a/b, where “a” represents the number of antibiotics to which the test isolate showed resistance, and “b” represents the total number of antibiotics used against that specific isolate in susceptibility testing [41]. All analyses were conducted at an alpha level of ≤0.05 with adjustment for multiple testing as mentioned above. Sensitivity analysis was performed to assess the robustness of the MAR index results by using the MAR index, organism type, location, total antibiotic resistance, and resistance profile variables (Table S5). In sensitivity analyses, the data were stratified by organism type and location to assess if resistance patterns are driven by these parameters. To assess whether extreme cases of the MAR index drive the results, indices ≥0.75 were removed from the analyses, and the full median MAR index dataset was compared with the median of the dataset without extreme MAR indices. Finally, a leave-one-species-out analysis was performed to assess whether a particular organism type disproportionally influenced the results.

#### 4.5.8. Ethical Approval

Ethical approval was obtained from the Department of Animal Health and Livestock (DAHLD) in Malawi (Ref: NO DAHLD/AHC/01/2025/02). Informed oral consent was obtained from every chicken vendor before sample collection. The researcher ensured that the vendors fully understood the study objectives, the sample collection process, their role during sample collection, and the usage of the data before obtaining consent.

## 5. Conclusions and Recommendations

This study reported high MAR indices of *E. coli*, *Salmonella* spp. and *Enterobacter* spp. from broiler meat sold in informal markets in Mzimba. This study identifies the need to train farmers on the proper usage of antibiotics in chicken production, including good management practices. There is a need to improve veterinary oversight both at the broiler farms and at markets, and surveillance of antimicrobial use and resistance among broiler farmers is needed. Similarly, vendors must also be trained on various programs such as proper hygiene practices when handling chicken meat, and there is also a need to regulate veterinary drug dispensing in Malawi.

## Figures and Tables

**Table 1:** *E. coli*, *Salmonella*, and *Enterobacter* species isolates’ resistance rates to antibiotics by location.

Location	Organism	CIP	AM	MEM	TCY	FEP	CRO	GEN	CHL
Area 1B	*E. coli*	33 (23)	100 (69)	100 (69)	100 (69)	0 (0)	11 (8)	55 (38)	33 (23)
	*Salmonella* spp.	0 (0)	0 (0)	100 (2)	0 (0)	0 (0)	0 (0)	100 (2)	0 (0)
Chibavi	*Enterobacter* spp.	0 (0)	100 (12)	100 (12)	100 (12)	100 (12)	0 (0)	0 (0)	0 (0)
	*E. coli*	0 (0)	100 (69)	100 (69)	80 (55)	60 (41)	0 (0)	0 (0)	20 (14)
Ekwendeni	*E. coli*	11(8)	89 (61)	89 (61)	66.7 (59)	89 (61)	0 (0)	0 (0)	44 (30)
Enukweni	*Enterobacter* spp.	0 (0)	100 (12)	100 (12)	100 (12)	0 (0)	0 (00	0 (0)	0 (0)
	*E. coli*	0 (0)	100 (69)	33 (23)	66.7 (59)	0 (0)	33 (23)	0 (0)	0 (0)
Kafukule	*Enterobacter* spp.	0 (0)	100 (12)	0 (0)	66.7 (8)	0 (0)	0 (0)	0 (0)	33 (4)
	*E. coli*	0 (0)	100 (69)	0 (0)	66.7 (59)	0 (0)	0 (0)	0 (0)	16.7 (16)
	*Salmonella*	0 (0)	100 (2)	0 (0)	100 (2)	0 (0)	0 (0)	0 (0)	0 (0)
Kavibale	*Enterobacter* spp.	0 (0)	100 (12)	100 (12)	100 (12)	0 (0)	0 (0)	0 (0)	0 (0)
	*E. coli*	20 (14)	100 (69)	80 (55)	100 (69)	20 (14)	0 (0)	20 (14)	20 (14)
Luwinga	*E. coli*	0 (0)	87.5 (60)	87.5 (60)	63 (43)	13 (9)	13 (9)	0 (0)	50 (35)
Mchengautuwa	*Enterobacter* spp.	0 (0)	50 (6)	100 (12)	50 (6)	0 (0)	0 (0)	0 (0)	50 (60
	*E. coli*	0 (0)	100 (69)	100 (69)	0 (0)	0 (0)	0 (0)	0 (0)	33 (23)
Mzuzu town	*Enterobacter* spp.	100 (12)	100 (12)	100 (12)	100 (12)	100 (12)	0 (0)	100 (12)	0
	*E. coli*	9 (6)	73 (50)	100 (69)	81 (56)	36 (25)	0 (0)	9 (6)	45 (31)
Nkholongo	*E. coli*	0 (0)	100 (69)	100 (69)	75 (52)	0 (0)	0 (0)	0 (0)	0 (0)
Zolozolo	*E. coli*	16.7 (12)	83 (57)	83 (57)	33 (23)	0 (0)	0 (0)	0 (0)	33 (23)

**Table 2:** Overall effect of organism type and location on MAR index.

Factor (Predictor)	Kruskal–Wallis Chi-Squared	df	*p*-Value	Effect Size Eta2 [η^2^] (Magnitude)
MAR by organism	3.967	2	0.1376	0.0246 (small)
MAR by location	36.813	10	˂0.0001 ***	0.372 (large)

***: sigificant.

**Table 3:** Beta regression analysis of selected predictors of the MAR index in broiler chicken samples.

Predictor	Category	Estimate	95% Confidence Interval		Std. Error	z Value	Pr (>|z|)
	(Intercept)	0.23023	−0.1800372	0.4102672	0.20932	1.100	0.271377
Location	Chibavi	−0.31244	−0.7613976	0.1365176	0.22906	−1.364	0.172569
	Ekwenden	−0.24249	−0.6313736	0.1463936	0.19841	−1.222	0.221646
	Enukweni	−0.90987	−1.4466944	−0.3730456	0.27389	−3.322	0.000894 ***
	Kafukule	−1.26340	−1.686956	−0.839844	0.21610	−5.846	5.02 × 10^−9^ ***
	Kaviwale	−0.47288	−0.9003756	−0.0453844	0.21811	−2.168	0.030157 *
	Luwinga	−0.57380	−0.9890456	−0.1585544	0.21186	−2.708	0.006762 **
	Mchengautuwa	−0.97962	−1.4888672	−0.4703728	0.25982	−3.770	0.000163 ***
	Mzuzu Town	−0.25864	−0.6313928	0.1141128	0.19018	−1.360	0.173843
	Nkholongo	−0.75244	−1.276624	−0.2282576	0.26744	−2.814	0.004900 **
	Zolozolo	−0.89979	−1.3635456	−0.4360344	0.23661	−3.803	0.000143 ***
Organism	*E. coli*	−0.09315	−0.397342	0.211042	0.15520	−0.600	0.54838
	*Salmonella* spp.	−0.80853	−1.5917264	−0.0253336	0.39959	−2.023	0.043033 *

* = less significant, ** = more significant, *** = highly significant.

**Table 4:** Disk diffusion breakpoints of *E. coli* and *Enterobacter* species.

Antibiotic Agent	Disk Content	Breakpoints for *E. coli* and *Enterobacter* spp. (Zone of Inhibition in mm)		
		Susceptible	Intermediate	Resistance
Ampicillin	10 μg	≥17	14–16	≤13
Tetracycline	30 μg	≥15	12–14	≤11
Chloramphenicol	10 μg	≥18	13–17	≤12
Ciprofloxacin	5 μg	≥26	22–25	≤21
Ceftriaxone	30 μg	≥23	20–22	≤19
Gentamicin	10 μg	≥18	15–17	≤14
Meropenem	10 μg	≥23	20–22	≤19
Cefepime	30 μg	≥25	19–24	≤18

**Table 5:** Disk diffusion breakpoints of *Salmonella* spp.

Antibiotic Agent	Disk Content	Breakpoints (Zone of Inhibition in mm)		
		Susceptible	Intermediate	Resistance
Ampicillin	10 μg	≥17	14–16	≤13
Tetracycline	30 μg	≥15	12–14	≤11
Chloramphenicol	10 μg	≥18	13–17	≤12
Ciprofloxacin	5 μg	≥31	21–30	≤20
Ceftriaxone	30 μg	≥26	23–25	≤22
Gentamicin	10 μg	≥15	13–14	≤12
Meropenem	10 μg	≥23	20–22	≤19
Cefepime	30 μg	≥26	22–25	≤21

## Data Availability

The majority of the data generated in this study are presented in the Results Section 2 through tables, figures and Supplementary Files. Additional data can be obtained from the corresponding authors upon reasonable request.

## Supplementary Materials

The following supporting information can be downloaded at: https://www.mdpi.com/article/10.3390/antibiotics15101028/s1, Figure S1. Q-Q plot indicating deviation of MAR index from reference line and stepwise clustering of values; Table S1: Multiple antibiotic resistance indices of the *E. coli* species and their corresponding antibiotics; Table S2: Multiple antibiotic-resistant indices for *Enterobacter* species and their corresponding antibiotics; Table S3: Wallis multiple comparison *p*-values adjusted with the Bonferroni method. Significant differences (*p*. adj < 0.05); Table S4: Shapiro-Wilk normality test results; Table S5: Results of the sensitivity analysis of the Multiple Antibiotic Resistance (MAR) index.

## Abbreviations and Acronyms

The following abbreviations are used in this manuscript:

AM	Ampicillin
AMR	Antimicrobial Resistance
AMRB	Antimicrobial-Resistant Bacteria
AMU	Antimicrobial Use
AST	Antibiotic Susceptibility Test
ATBR	Antimicrobial Bacteria Resistance
CHL	Chloramphenicol
CIP	Ciprofloxacin
CLSI	Clinical Laboratory Standard Institute
CRO	Ceftriaxone
DNA	Deoxynucleic Acid
EU	European Union
FAO	Food and Agriculture Organization
FAS	Food Animal Source
GEN	Gentamycin
IFT	Implications for the Food System
KAP	Knowledge, Attitudes and Practices
LMICs	Low- and Middle-Income Countries
MDR	Multidrug Resistance
MEM	Meropenem
MARI	Multiple Antibiotic Resistant Index
Spp	Species
TET	Tetracycline
TSB	Trypticase Soy Broth
WHO	World Health Organization
