# Supplementary material for: Phenotypic Antimicrobial Resistance Patterns in E. coli, Salmonella, and Enterobacter Species Isolated from Broiler Chicken Meat in Mzimba, Malawi: Insights from the Multiple Antibiotic Resistance Index
Source: Antibiotics (Basel). 2026 Oct 16;15(10):1028. doi: 10.3390/antibiotics15101028 (PMC13700800; doi:10.3390/antibiotics15101028)
Supplement: Supplementary file 1 [file antibiotics-15-01028-s001.zip › antibiotics-4429119-supplementary.pdf]

Supplementary material

1. Multiple Antibiotic Resistance Index (MAR Index)

Table S1: Multiple antibiotic resistance indices of the *E. coli* species and their corresponding antibiotics

MAR	**TOTAL ISOLATES**	**ANTIBIOTICS**	**P-value**
0.75	2	CIP,AM,MEM,TCY,GEN,CHL	0.001
0.63	2	AM.MEM,TCY,FEP,CHL	
0.63	1	AM,MEM,TCY,CRO,GEN	
0.63	1	CIP,AM,MEM,FEP,CHL	
0.63	2	CIP,AM,MEM,TCY,GEN	
0.5	7	AM,MEM,TCY,FEP	
0.5	3	AM,MEM,TCY,CHL	
0.5	2	AM,MEM,TCY,GEN	
0.5	2	CIP,AM,MEM,CHL	
0.5	1	AM,MEM,TCY,GEN	
0.5	1	MEM,TCY,FEP,CHL	
0.5	1	AM,MEM,TCY,CRO	
0.5	1	AM, MEM,FEP, CHL	
0.4	14	AM,MEM,TCY	
0.4	4	AM,MEM,CHL	
0.4	2	AM,TCY,CHL	
0.4	2	AM,MEM,FEP	
0.4	2	MEM,TCY,CHL	
0.4	1	AM,TCY,FEP	
0.4	1	MEM,FEP,CHL	
0.4	1	MEP, TCY, FEP	
0.25	6	AM,TCY	
0.25	7	AM,MEM	
0.25	1	MEM,TCY	
0.25	1	MEM,CHL	
0.1	1	AM	

Table S2: Multiple antibiotic-resistant indices for *Enterobacter* species and their corresponding antibiotics

MAR	Total isolates	Antibiotics
0.75	1	CIP, AM, MEM, TCY, FEP, GEN
0.5	1	AM, MEM, TCY, FEP
0.375	5	AM, MEM, TCY
0.375	1	MEM, TCY, FEP
0.375	1	AM, TCY, CHL
0.25	1	AM, TCY
0.25	1	MEM, CHL
0.125	1	AM

2. Inferential statistics supplementary materials

Table S3: Dunn (1964), Kruskal-Wallis multiple comparison p-values adjusted with the Bonferroni method. Significant differences (P. adj < 0.05)

No	Comparison	Z	P. unadj	P. adj
1	Area 1b - Chibavi	-0.104	0.917	1.000
2	Area 1b - Ekwenden	-0.311	0.756	1.000
3	Chibavi - Ekwenden	-0.166	0.868	1.000
4	Area 1b - Enukweni	2.159	0.031*	1.000
5	Chibavi - Enukweni	2.062	0.039*	1.000
6	Ekwenden - Enukweni	2.395	0.017*	0.916
7	Area 1b - Kafukule	3.951	<0.001***	0.004**
8	Chibavi - Kafukule	3.525	<0.001***	0.023*
9	Ekwenden - Kafukule	4.262	<0.001***	0.001**
10	Enukweni - Kafukule	0.828	0.408	1.000
11	Area 1b - Kaviwale	1.007	0.314	1.000
12	Chibavi - Kaviwale	0.984	0.325	1.000
13	Ekwenden - Kaviwale	1.301	0.193	1.000
14	Enukweni - Kaviwale	-1.306	0.192	1.000
15	Kafukule - Kaviwale	-2.718	0.007**	0.361
16	Area 1b - Luwinga	1.191	0.234	1.000
17	Chibavi - Luwinga	1.145	0.252	1.000
18	Ekwenden - Luwinga	1.484	0.138	1.000
19	Enukweni – Luwinga	-1.164	0.245	1.000
20	Kafukule - Luwinga	-2.535	0.011*	0.619
21	Kaviwale - Luwinga	0.174	0.862	1.000
22	Area 1b - Mchengautuwa	2.543	0.011*	0.605
23	Chibavi - Mchengautuwa	2.388	0.017*	0.931

24	Ekwenden - Mchengautuwa	2.797	0.005**	0.284
25	Enukweni - Mchengautuwa	0.172	0.864	1.000
26	Kafukule - Mchengautuwa	-0.68	0.494	1.000
27	Kaviwale - Mchengautuwa	1.605	0.109	1.000
28	Luwinga - Mchengautuwa	1.452	0.146	1.000
29	Area 1b - Mzuzu Town	0.218	0.828	1.000
30	Chibavi - Mzuzu Town	0.294	0.789	1.000
31	Ekwenden - Mzuzu Town	0.542	0.587	1.000
32	Enukweni - Mzuzu Town	-2.051	0.040*	1.000
33	Kafukule - Mzuzu Town	-3.909	0.000***	0.005**
34	Kaviwale - Mzuzu Town	-0.842	0.400	1.000
35	Luwinga - Mzuzu Town	-1.033	0.302	1.000
36	Mchengautuwa - Mzuzu Town	-2.441	0.015*	0.805
37	Area 1b - Nkholongo	1.672	0.095	1.000
38	Chibavi - Nkholongo	1.615	0.106	1.000
39	Ekwenden - Nkholongo	1.907	0.057	1.000
40	Enukweni - Nkholongo	-0.408	0.683	1.000
41	Kafukule - Nkholongo	-1.315	0.189	1.000
42	Kaviwale - Nkholongo	0.835	0.404	1.000
43	Luwinga - Nkholongo	0.693	0.488	1.000
44	Mchengautuwa - Nkholongo	-0.602	0.547	1.000
45	Mzuzu Town - Nkholongo	1.551	0.121	1.000
46	Area 1b - Zolozolo	2.480	0.013*	0.722
47	Chibavi - Zolozolo	2.312	0.020*	1.000
48	Ekwenden - Zolozolo	2.750	0.001*	0.328
49	Enukweni - Zolozolo	0.006	0.996	1.000
50	Kafukule - Zolozolo	-0.941	0.347	1.000
51	Kaviwale - Zolozolo	1.487	0.137	1.000
52	Luwinga - Zolozolo	1.326	0.185	1.000
53	Mchengautuwa – Zolozolo	-0.185	0.854	1.000
54	Mzuzu Town - Zolozolo	2.375	0.018*	0.964
55	Nkholongo - Zolozolo	0.452	0.651	1.000

3. Normality test results

Table S4: Shapiro-Wilk normality test results

W-statistic	P-value
**0.89436**	< 0.001***

The W-statistic is far from 1, indicating that the data is not normally distributed, and the p-value is significant.

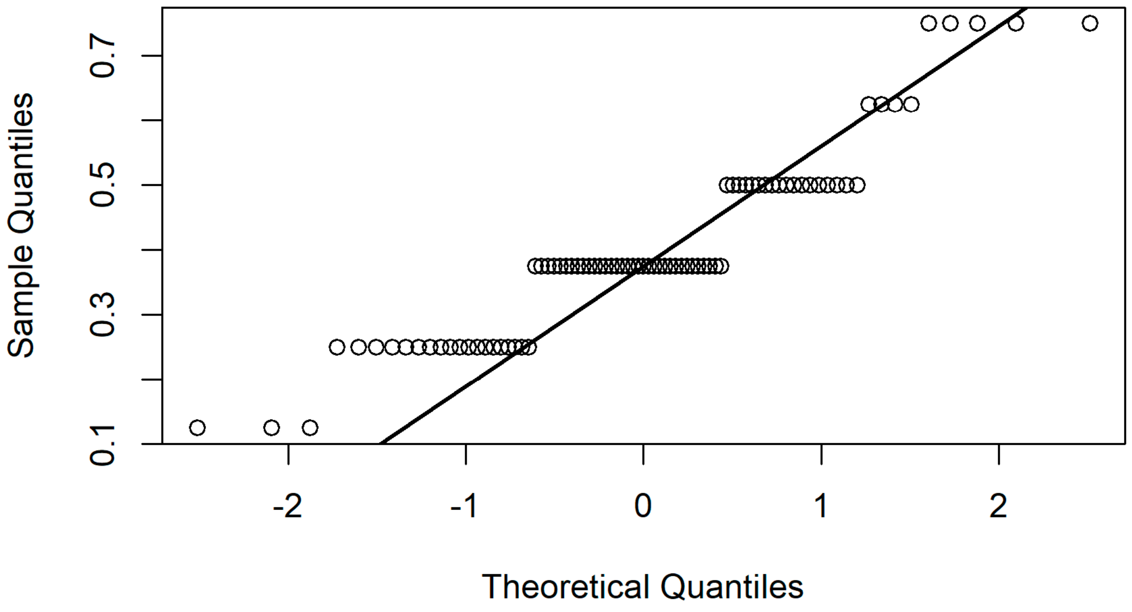

Figure S1. Q-Q plot indicating deviation of MAR index from reference line and stepwise clustering of values.

4. Sensitivity analysis

Table S5: Results of the sensitivity analysis of the Multiple Antibiotic Resistance (MAR) index

Method	Median	Explanation
All isolates (n = 83)	0.375	Overall median MAR across all isolates
Excluding high MAR isolates (≥0.75) (n = 76)	0.375	Tested the influence of extreme resistance profiles
Leave-one-species-out: Excluding *E. coli* (n = 71)	0.375	Assessed the effect of removing *E. coli*
Leave-one-species-out: Excluding *Enterobacter* (n = 12)	0.375	Assessed the effect of removing *Enterobacter*
Leave-one-species-out: Excluding *Salmonella* (n = 81)	0.375	Assessed the effect of removing *Salmonella*

Notes: The median remained stable under all sensitivity testing methods, indicating robustness of the results.
